# Lambda-carrageenan treatment exacerbates the severity of cerebral malaria caused by *Plasmodium berghei* ANKA in BALB/c mice

**DOI:** 10.1186/1475-2875-13-487

**Published:** 2014-12-11

**Authors:** Frances C Recuenco, Ryo Takano, Shiori Chiba, Tatsuki Sugi, Hitoshi Takemae, Fumi Murakoshi, Akiko Ishiwa, Atsuko Inomata, Taisuke Horimoto, Yoshiyasu Kobayashi, Noriyuki Horiuchi, Kentaro Kato

**Affiliations:** Department of Veterinary Microbiology, Graduate School of Agricultural and Life Sciences, The University of Tokyo, 1-1-1 Yayoi, Bunkyo-ku, Tokyo, 113-8657 Japan; National Research Center for Protozoan Diseases, Obihiro University of Agriculture and Veterinary Medicine, Obihiro 080-8555, Hokkaido, Japan; Department of Basic Veterinary Medicine, Obihiro University of Agriculture and Veterinary Medicine, Obihiro 080-8555, Hokkaido, Japan; Department of Veterinary Paraclinical Sciences, College of Veterinary Medicine, University of the Philippines Los Baños, Laguna, 4031 Philippines

**Keywords:** Cerebral malaria, λ-carrageenan, BALB/c mouse, Intracerebral haemorrhage, *Plasmodium berghei* ANKA

## Abstract

**Background:**

There is an urgent need to develop and test novel compounds against malaria infection. Carrageenans, sulphated polysaccharides derived from seaweeds, have been previously shown to inhibit *Plasmodium falciparum in vitro*. However, they are inflammatory and alter the permeability of the blood–brain barrier, raising concerns that their use as a treatment for malaria could lead to cerebral malaria (CM), a severe complication of the disease. In this work, the authors look into the effects of the administration of λ-carrageenan to the development and severity of CM in BALB/c mice, a relatively non-susceptible model, during infection with the ANKA strain of *Plasmodium berghei*.

**Methods:**

Five-week-old female BALB/c mice were infected with *P. berghei* intraperitoneally. One group was treated with λ-carrageenan (PbCGN) following the 4-day suppressive test protocol, whereas the other group was not treated (PbN). Another group of healthy BALB/c mice was similarly given λ-carrageenan (CGN) for comparison. The following parameters were assessed: parasitaemia, clinical signs of CM, and mortality. Brain and other vital organs were collected and examined for gross and histopathological lesions. Evans blue dye assays were employed to assess blood–brain barrier integrity.

**Results:**

*Plasmodium berghei* ANKA-infected BALB/c mice treated with λ-carrageenan died earlier than those that received no treatment. Histopathological examination revealed that intracerebral haemorrhages related to CM were present in both groups of infected BALB/c mice, but were more numerous in those treated with λ-carrageenan than in mock-treated animals. Inflammatory lesions were also observed only in the λ-carrageenan-treated mice. These observations are consistent with the clinical signs associated with CM, such as head tilt, convulsions, and coma, which were observed only in this group, and may account for the earlier death of the mice.

**Conclusion:**

The results of this study indicate that the administration of λ-carrageenan exacerbates the severe brain lesions and clinical signs associated with CM in BALB/c mice infected with *P. berghei* ANKA*.*

## Background

Carrageenans are linear sulphated polysaccharides derived from seaweeds. The three types of carrageenans are characterized according to their levels of sulphation and are identified by Greek letters: κ-carrageenan, ι-carrageenan, and λ-carrageenan. κ-carrageenan has one sulphate group and forms rigid gels, ι-carrageenan has two sulphate groups and forms soft gels, and λ-carrageenan has three sulphate groups and does not gel in solution.

Carrageenans have known anti-malarial activity *in vitro* and *in vivo*. Adams *et al.* previously demonstrated that carrageenans inhibit the growth and invasion of *Plasmodium falciparum* 3D7 and Dd2 *in vitro* with λ-carrageenan having the highest inhibitory activity, followed by ι-carrageenan, and κ-carrageenan [1]. Further, James *et al.* reported that calcium carrageenan pre-treatment of A/J mice decreased the parasitaemia and delayed death from infection with the NK65 strain of *Plasmodium berghei* [2].

Carrageenans are common additives in food and personal hygiene products such as toothpastes and personal lubricants. However, the use of carrageenans as additives in foods such as meat and milk has become controversial. Several studies have linked carrageenans to inflammatory bowel disease [3] and to the development of several allergies [4, 5] and even mammary carcinoma [6]. Carrageenans have been used to induce inflammation in inflammatory models. Carrageenan-induced inflammation in the paw of mice or rats is a classic model of oedema formation and hyperalgesia that is useful in the development of non-steroidal anti-inflammatory drugs. Among the three types of carrageenan, κ-carrageenan was found to be the most inflammatory in the paw oedema model, whereas λ-carrageenan had the highest anticoagulant activity *in vitro* [7].

Carrageenan-induced inflammation involves the release of histamine and serotonin followed by the release of prostaglandins, protease, and lysosomes, producing oedema [8]. Huber *et al.* showed that 72-h exposure of rats to λ-carrageenan alters the permeability of the blood–brain barrier [9]. Given that dysfunction of the blood–brain barrier has been specifically observed in cerebral malaria, carrageenans could cause this serious side effect if used as anti-malarial drugs in clinical practice.

Human cerebral malaria (HCM) is the most serious and often fatal complication of severe *P. falciparum* malaria. Patients inflicted with HCM become comatose and show neurological symptoms, including seizures, retinopathy, brainstem alterations, and brain swelling. Those who survive HCM develop long-term neurological sequelae, as well as cognition and behavioural deficits [10].

Although the pathogenesis of HCM is still poorly defined, the main cause is thought to involve the sequestration of infected red blood cells (iRBC) in brain capillaries [11]. Leukocytes and platelets get sequestered on the endothelium, which leads to occlusion of brain capillaries and poor microvascular flow, decreased nutrient supply to the brain, and damage to vascular walls that ultimately leads to haemorrhages and neuronal alterations [12]. Sequestration is a characteristic of *P. falciparum* malaria that involves molecular adhesion between parasite proteins, such as PfEMP1, the knob protein expressed on the surface of infected erythrocytes, and ligands on endothelial cells, such as CD36, thrombospondin, and ICAM-1 [13].

Experimental cerebral malaria (ECM) can be demonstrated by using the rodent malaria model *P. berghei* ANKA in susceptible mouse strains, such as C57BL/6 mice. However, the use of this ECM mouse model highly controversial because not all aspects of HCM can be reproduced. The most accepted mechanism underlying the development of ECM involves the accumulation of inflammatory cells in the brain, a characteristic of encephalitis [14], rather than sequestration of infected red blood cells.

Here, the authors investigate whether administration of λ-carrageenan can contribute to the development of cerebral malaria in BALB/c mice, a relatively non-susceptible model of experimental CM during infection with the ANKA strain of *P. berghei.* Histopathological analyses were also performed and the severity of the brain lesions in infected-mice treated with λ-carrageenan was evaluated.

## Methods

### Ethics statement

The mice used in the study were maintained in the animal care facility of NRCPD, Obihiro University of Agriculture and Veterinary Medicine under controlled conditions and were given commercial feeds and water *ad libitum*. The protocol for the experiments was approved by the Committee on the Animal Experiments of the Obihiro University of Agriculture and Veterinary Medicine (Approval number: 25–153).

### Parasites and animals

Frozen stock of *P. berghei* ANKA parasites were provided by Dr. Motomi Torii (Ehime University), and were passaged in BALB/c mice. Female BALB/c mice, five weeks of age, were purchased from CLEA Japan (Tokyo, Japan) and used in the study.

### Infection and treatment

Donor mice were infected intraperitoneally with 10^6^ parasitized red blood cells from a cryopreserved stock. When the parasitaemia reached around 15%, infected blood was collected by cardiac puncture and mixed with PBS. Two groups of experimental mice were infected intraperitoneally with 10^7^ parasitized red blood cells: the first group was the untreated group (PbN group) and the other group was the carrageenan-treated group (PbCGN group). Another group of uninfected mice was also treated with λ-carrageenan (CGN group). λ-carrageenan (SIGMA) was dissolved in PBS (2 mg/ml) at 65°C and passed through a 0.45-μm filter (Millipore) for sterilization prior to use. λ-carrageenan was given intraperitoneally at a dose of 25 mg/kg BW following the four-day suppressive test. In this protocol, the infection day is designated as day 0. The first treatment is given 2 h after infection, and then subsequent treatments are given every 24 h until day 3 post-infection, for a total of four treatments.

To determine the survival rate, half of the infected mice in each group were observed for clinical signs of cerebral malaria until the mice eventually succumbed to the disease. The other half of each group was sacrificed under terminal isoflurane anesthesia at the presumed onset of cerebral malaria for histopathological examination and Evans blue dye assays.

### Parasitaemia, ECM signs, and survival monitoring

Parasitaemia levels of both groups of infected mice (n = 4) were monitored from days 4 to 10 post-infection by using Giemsa-stained thin blood smears. Ten oil immersion objective fields were examined each with 200–300 red blood cells. The infected mice were observed for general signs of malaria such as ruffled fur, and hunching posture, and for ECM-related signs such as wobbly gait, head tilt, limb paralysis, convulsions, and coma. Other behavioural parameters monitored included reactions to stimuli such as exploration of a new environment and touch reflexes [15, 16]. Deaths were promptly recorded.

### Histopathology, intracerebral haemorrhage scoring, and statistical analysis

The infected mice were sacrificed under terminal isoflurane anesthesia once the clinical signs of ECM were observed in some or all members of the treatment groups. This corresponded to day 5 p.i. in all members of the PbCGN group (n = 4). Although no signs of CM were observed, the PbN mice (n = 4) were also sacrificed at this time. Normal and healthy mice (N, n = 4) and uninfected, carrageenan-treated mice (CGN, n = 4) were also sacrificed at the same time for comparison. Brains as well as other vital organs including kidneys, livers, hearts, lungs, and spleens were collected and fixed in 4% paraformaldehyde and embedded in paraffin. The organs were sectioned at 5 μm and were stained with haematoxylin-eosin (HE) stain. The brain sections were examined for haemorrhages and inflammation. Intracerebral haemorrhages were recorded [16]. Statistically significant differences in the number of intracerebral haemorrhages were determined by using the Mann–Whitney U test. Values were considered to be significantly different when the *P* value was less than 0.05. The organ sections were examined for any significant lesions.

### Assessment of vascular leakage at the blood–brain barrier by use of Evans blue dye perfusion

On day 5 post-infection, 200 μl of 1% Evans blue dye was injected into the tail veins of representative mice (n = 3–4) from each treatment group. After 1 h, the mice were sacrificed and their brains were collected and examined for bluish discoloration. The brains were then placed in 4% paraformaldehyde for 48 h to extract the Evans blue dye, and the absorbance was measured at a wavelength of 600 nm [15]. For this, the results of two independent assays were pooled. Absorbance readings were carried out in triplicates and the mean of three replicates was computed. Statistically significant differences in the absorbance of Evans blue dye between the groups were determined by using Tukey’s multiple comparison test. Values were considered to be significantly different when the *P* value was less than 0.05.

## Results

### Effects of λ-carrageenan treatment on parasitaemia and course of infection

**Figure 1:**
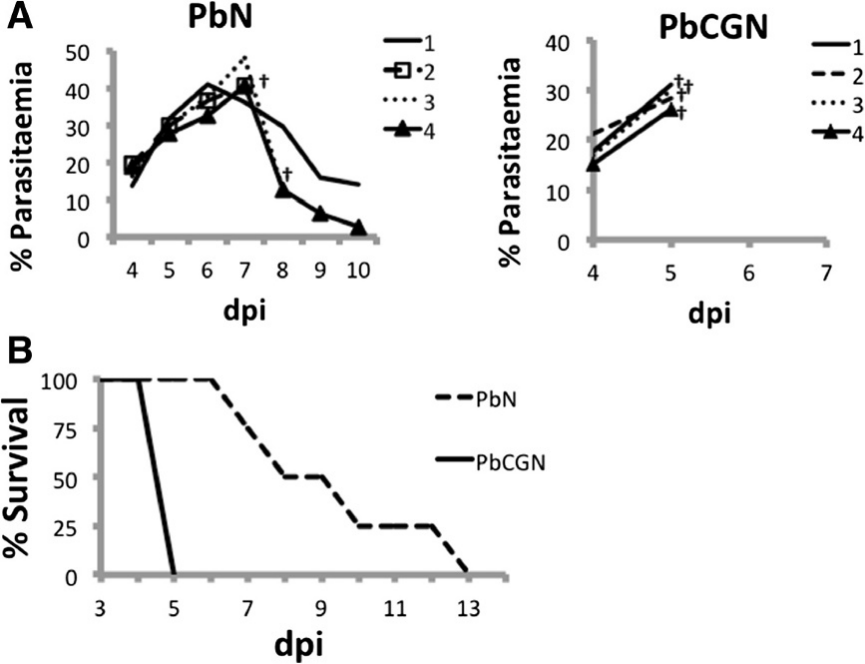
**Parasitaemia and survival profiles of** ***P. berghei*** **ANKA-infected BALB/c mice. (A)** Four mice per group were infected with *P. berghei* ANKA, and were not treated (PbN) or treated with λ-carrageenan (PbCGN). The parasitaemia in the blood microvessels is shown for each animal per group (n = 4). λ-carrageenan treatment (25 mg/kg) did not inhibit the growth of *P. berghei* ANKA *in vivo* using the four-day suppressive test. The dagger (†) indicates mouse death. **(B)** Survival profiles of *P. berghei* ANKA-infected BALB/c mice: λ-carrageenan-treated (PbCGN); not treated (PbN).

Lambda-carrageenan poorly inhibited the growth of *P. berghei* ANKA, as shown by the hyperparasitaemia in the infected mice (Figure 1A), and did not improve the survival of the infected mice (Figure 1B). These findings differ from the report by James and Alger [2], who found that A/J Swiss mice infected with *P. berghei* NK65 survived for up to 28 days when pre-treated with calcium carrageenan intraperitoneally, and from the finding of Adams that λ-carrageenan effectively inhibited the growth and invasion of red blood cells by *P. falciparum* in *in vitro* experiments [1].

In the experiments monitoring parasitaemia, ECM, and survival, all *P. berghei* ANKA-infected mice exhibited signs of ruffled fur, hunching, and decreased reaction to stimuli on day 5 post-infection. PbCGN mice showed limb paralysis, convulsions, head tilt, and coma and died soon afterwards. On the same day, PbN mice showed none of these neurological signs. Deaths in the PbN mice group were first seen on day 7 p.i and all of the PbN mice died by day 13 p.i. Signs observed in the PbN mice that started to die from day 7 until day 13 p.i. included general weakness and lethargy, while signs that are related to ECM, specifically, limb paralysis, convulsions, and head tilt were not seen.

### Gross pathology and histopathology of the brains of *Plasmodium berghei*-infected BALB/c mice

**Figure 2:**
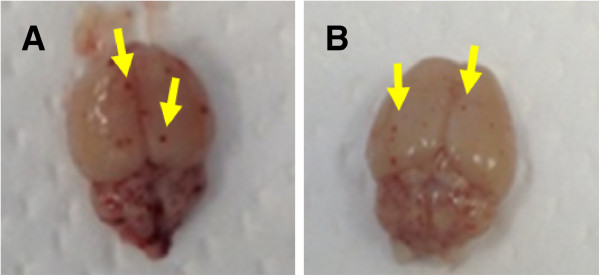
**Gross lesions on the brains of infected mice. (A)** From PbCGN mice that were sacrificed at the presumed onset of cerebral malaria 5 days p.i. showing clinical signs such as head tilting and convulsions. **(B)** From a Pb-infected, untreated (PbN) mouse sacrificed on day 5 p.i. that did not show any signs associated with CM. Yellow arrows show petechial haemorrhages.

Haemorrhages were visible on the brains of the PbCGN mice that showed clinical signs of CM and were sacrificed on day 5 p.i. (n = 4) (Figure 2A). Similarly, haemorrhages were also observed in the PbN mice that were sacrificed on the same day (n = 4) (Figure 2B). These PbN mice, however, did not necessarily show neurological signs related to cerebral malaria. This result shows that in the BALB/c mouse, which is considered to be resistant to the development of CM by *P. berghei* ANKA infection, in the absence of clear neurological signs, haemorrhagic brain lesions can be observed.

**Figure 3:**
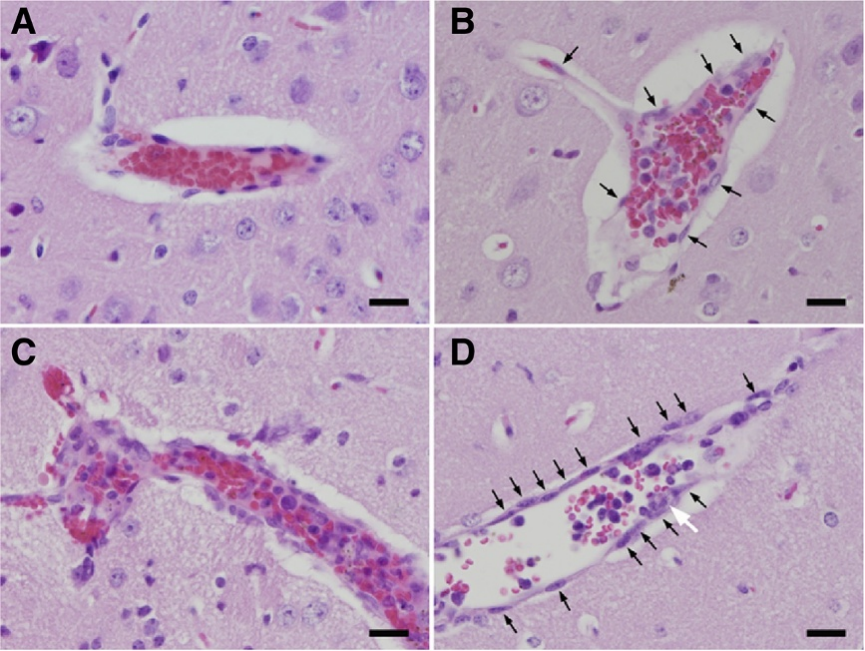
**Brain histopathology. (A)** CGN group. No apparent lesions are observed (Haematoxylin-Eosin stain). Bar, 20 μm. **(B)** PbN group. Microthrombus consisting of fibrin pigmented RBCs and accumulated mononuclear cells, is observed. Perivascular infiltration of inflammatory cells is not observed. Vascular endothelium is not hyperplastic (arrows indicate endothelial cells). Bar, 20 μm. **(C)** PbCGN group. Microthrombus consisting of fibrin, pigmented RBCs, pigmented macrophages, and mononuclear cells is observed. Perivascular infiltration of inflammatory cells, haemorrhage in perivascular space, and hyperplastic vascular endothelium are also observed. Bar, 20 μm. **(D)** PbCGN group. Infected RBCs and inflammatory cells can be seen within the blood vessels of the cerebrum. Note the hyperplastic endothelium, characterized by increased number of endothelial cells (arrow). Adhesive Pb-laden macrophages (shown by white arrow) can also be seen. Bar, 20 μm.

To further characterize the lesions in the brains of mice infected with *P. berghei* ANKA and then treated with λ-carrageenan, two independent experiments for histopathological examinations were done with similar observations. No apparent lesions were observed in the brain sections of uninfected, healthy carrageenan-treated mice (CGN group, Figure 3A). By contrast, microthrombi, intracerebral haemorrhages, and the presence of iRBCs in brain vessels were observed in both PbN (as shown in Figure 3B) and PbCGN (Figure 3C and D) animals. Perivascular infiltrations of inflammatory cells around the microthrombi were also observed in the PbCGN group and not as much in the PbN group. In addition, hyperplastic endothelium of brain blood vessels was observed only in the PbCGN group (Figure 3C and D). These results indicate that the administration of λ-carrageenan to BALB/c mice infected with *P. berghei* ANKA caused more severe histopathological features associated with cerebral malaria.

Histopathology of other vital organs revealed malaria pigment deposits in lung sections and in the Kupffer cells in the livers of both the PbN and PbCGN groups. The presence of necrotized lymphocytes and follicular hyperplasia was also observed in the spleens of both infected groups. No apparent lesions were observed in the kidneys or hearts of these infected mice. These results show that λ-carrageenan treatment had no atypical effects on the other vital organs of the *P. berghei*-infected BALB/c mice.

### Intracerebral haemorrhage counts

Intracerebral haemorrhages observed in sections from four major regions of the brain, namely, the frontal lobe, diencephalon, occipital lobe, and cerebellum, were counted and tallied (n = 4, PbN and PbCGN, respectively). There were no statistically significant differences in the number of intracerebral haemorrhages between the PbN and PbCGN groups. However, haemorrhages were observed in all regions of the brains of all of the PbCGN mice, whereas they were observed in only half of the PbN mice tested (Table 1). These findings are consistent with the increased severity of the pathology in the PbCGN mice relative to the PbN mice.

**Table 1:** **Intracerebral haemorrhage counts**

Haemorrhage score	PbN				PbCGN				Mann–Whitney ***P***
	A	B	C	D	E	F	G	H	
**Frontal Lobe**	**2**	**0**	**0**	**4**	**6**	**4**	**9**	**3**	**0.05755**
**Diencephalon**	**5**	**0**	**0**	**1**	**6**	**4**	**9**	**3**	**0.1059**
**Occipital Lobe**	**4**	**2**	**0**	**6**	**2**	**4**	**7**	**2**	**0.6552**
**Cerebellum**	**5**	**0**	**0**	**1**	**4**	**6**	**4**	**4**	**0.1367**
**Total**	**16**	**2**	**0**	**12**	**14**	**19**	**27**	**11**	**0.1489**

Statistically significant differences in intracerebral haemorrhage counts were determined by using the Mann–Whitney test. Values were considered to be significantly different when the *P* value was less than 0.05.

### Assessment of vascular leakage at the blood–brain barrier by using Evans blue dye perfusion

**Figure 4:**
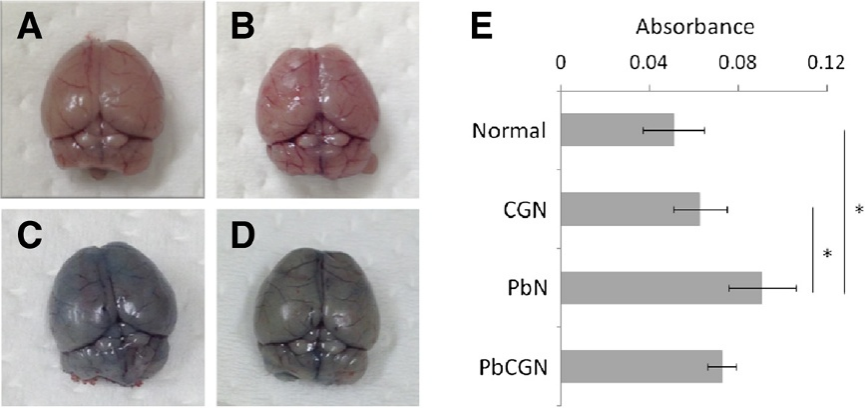
**Assessment of vascular leakage at the blood–brain barrier by use of Evans blue dye perfusion.** Two hundred microliters of 1% Evans blue dye was injected into the tail veins of each mice on day 5 post-infection. After 1 h, the mice were sacrificed using terminal isoflurane anesthesia and the brains were collected. Representative brains are shown: **(A)** Normal healthy mouse (N), **(B)** Carrageenan-treated mouse (CGN), **(C)** *P. berghei* ANKA-infected, untreated mouse (PbN), **(D)** *P. berghei* ANKA-infected, carrageenan-treated mouse (PbCGN). **(E)** The mean absorbances at 600 nm wavelength and standard deviations of extracted dye after placing the brains in 4% paraformaldehyde for 48 h are shown for each group. Statistically significant differences in mean absorbances were determined by using Tukey’s multiple comparison test (*: *P* value < 0.05, ns: not significant).

To further evaluate the effects of λ-carrageenan on the integrity of blood–brain barrier (BBB), and its contribution to the early death of the mice infected with *P. berghei* ANKA, the permeability of the blood–brain barrier was assessed by using Evans blue assay. Representative images of the brains of mice from each group are shown in Figure 4. The brains of the PbN and PbCGN mice (Figure 4C and D, respectively) absorbed the dye, indicating increased permeability of the blood–brain barrier compared with that of the brains of normal mice and CGN mice (Figure 4A and B, respectively). As expected, CGN mice showed increased levels of blood–brain barrier leakage relative to the levels in the brains of normal mice. However, there were no statistically significant differences in the levels of Evans blue dye leakage from the mouse brains between the PbN and PbCGN groups (Figure 4E). These results indicate that not only the altered integrity of blood–brain barrier but also other factors may lead to this severe complication of malaria in mice infected with *P. berghei* ANKA.

## Discussion

Here, the *in vivo* efficacy and safety of λ-carrageenan as an anti-malarial drug was evaluated. The results show that λ-carrageenan poorly inhibited the growth of *P. berghei* ANKA in BALB/c mice and caused more severe brain lesions, leading to the early death of the mice. BALB/c mice, which are considered to be relatively resistant to the consequences of cerebral malaria caused by the ANKA strain of *P. berghei* [10, 17–19], were chosen as model to assess the effects of the administration of λ-carrageenan to the development of clinical signs associated with cerebral malaria. Both groups were shown to develop a high parasitaemia, but the infected BALB/c mice treated with λ –carrageenan showed more signs related to ECM and died earlier than the untreated mice. The attempts to show that λ-carrageenan treatment could cause more severe cerebral malaria through histopathology and Evans blue dye assays revealed almost the same results for both groups of infected mice. The levels of blood–brain barrier leakage were similar between the *P. berghei*-infected mice that were mock-treated and those that were administered λ-carrageenan; and the histopathological findings on the brains show the presence of intracranial haemorrhages for both groups. It is important to note that with the small sample size used in this study, the statistical data presented here should be interpreted with caution [20–22].

It is also possible that although the clinical signs associated with ECM in the untreated group were not observed, these mice may also have developed ECM, as reported by Neill and Hunt [23]. It will be interesting to explore the underlying mechanisms as to why clear neurological signs were not as evident in the untreated group given that there was hyperparasitaemia, leakage in the BBB and presence of intracranial haemorrhages. These results indicate that there may be other factors involved in the early death of mice treated with λ-carrageenan during *P. berghei* ANKA infection. One can also consider the possibility that the hyperplastic endothelium of the brain blood vessels compensated for the blood–brain barrier leakage in the PbCGN group.

Lambda-carrageenan is known to induce inflammatory pain and to alter the permeability of the blood–brain barrier in several animal models [9, 24]. Moreover, administration of λ-carrageenan increases the expression of ICAM-1, which plays a key role in the sequestration of iRBCs in cerebral malaria under experimental and natural conditions [25, 26].

By contrast, λ-carrageenan is known to activate the innate immunity that depends on Toll-like receptor 4 (TLR4) and Myd88 [5]. Given that the activation of TLR4/Myd88 signaling causes acute inflammatory injury [27], one could argue that the early deaths of mice treated with λ-carrageenan during the *P. berghei* ANKA infection could be attributed to the dysregulation of innate immunity rather than the dysfunction of the blood–brain barrier. Further studies are warranted to elucidate the molecular basis of this exacerbation of pathology.

Malaria remains a threat to human health and there is an urgent need to develop and test novel compounds. The results presented here suggest that λ-carrageenan, a sulphated polysaccharide which is a common food additive and ingredient in household products and has been found to inhibit the invasion of red blood cells by malaria merozoites *in vitro*, may be unsuitable for the treatment of clinical malaria.

However, other sulphated polysaccharides, including heparin, fucoidan, and dextran sulfate [28, 29] have shown promising *in vitro* anti-malarial activity. An example is the previous report on gellan sulfate [30] that demonstrated that chemical modification, namely, sulphation, could change the characteristics of these compounds. Given that these polysaccharides are still considered promising potential anti-malaria drugs, chemical modification might decrease the side effects of such compounds.

## Conclusion

This study shows the potential toxicity of λ-carrageenan as an antimalarial using the BALB/c mice as model of ECM. We find the usefulness of this rodent model in elucidating CM pathogenesis and evaluating promising antimalarial candidates *in vivo* and more importantly the safety profile of anti-malarial compounds that could not be envisaged if only *in vitro* experiments were conducted.

## Abbreviations

CM: Cerebral malaria
ECM: Experimental cerebral malaria
HCM: Human cerebral malaria
BBB: Blood–brain barrier
iRBCs: Infected red blood cells
H-E: Haematoxylin-eosin stain.
